# Design and optimization of four-terminal mechanically stacked and optically coupled silicon/perovskite tandem solar cells with over 28% efficiency

**DOI:** 10.1016/j.heliyon.2023.e13477

**Published:** 2023-02-03

**Authors:** Ehsan Raza, Zubair Ahmad, Fakhra Aziz, Muhammad Asif, Muhammad Qasim Mehmood, Jolly Bhadra, Noora J. Al-Thani

**Affiliations:** a Department of Electronics, University of Peshawar, Peshawar, 25120, Pakistan; b Qatar University Young Scientists Center (QUYSC), Qatar University, 2713, Doha, Qatar; c MicroNano Lab, Electrical Engineering Department, Information Technology University (ITU) of the Punjab, Ferozepur Road, Lahore 54600, Pakistan

**Keywords:** HTM-Free perovskite PV cells, Crystalline silicon solar cells, Mechanically stacked four-terminal tandem, Optically coupled four-terminal tandem, SCAPS-1D, Energy conversion efficiency

## Abstract

Silicon/perovskite tandem devices are believed to be a favorite contender for improving cell performance over the theoretical maximum value of single-junction photovoltaic (PV) cells. The present study evaluates the design and optimization of four-terminal (4-T) mechanically stacked and optically coupled configurations using SCAPS (solar cell capacitance simulator). Low-cost, stable, and easily processed semitransparent carbon electrode-based perovskite solar cells (c-PSCs) without hole transport material (HTM) and highly efficient crystalline silicon (c-Si) PV cells were utilized as top and bottom cells, respectively. The wide bandgap multi-cation perovskite ${Cs}_{x}{\left({FA}_{0.4}{MA}_{0.6}\right)}_{1-x}{PbI}_{2.8}{Br}_{0.2}$ and a low bandgap c-Si were employed as light-harvesting layers in the top and bottom cells, respectively. The impact of perovskite thickness and doping concentrations were examined and optimized for both tandem configurations. Under optimized conditions, thicknesses of 1000 nm and 1100 nm are the best values of the perovskite absorber layer for 4-T mechanically stacked and optically coupled arrangements, respectively. Likewise, 1 × 10^17^ cm^−3^ doping concentration of top cells revealed the highest performance in both structures. With these optimized parameters under tandem configurations, efficiency values of 28.38% and 29.34% were obtained in 4-T mechanically and optically coupled tandems, respectively. Results suggest that by optimizing perovskite thickness and doping concentration, the proposed designs using HTM-free c-PSCs could enhance device performance.

## 1 Introduction

The photovoltaic (PV) industry has been overtaken by crystalline silicon (c-Si) PV cells, with a market share of over 95% [1]. The c-Si cells are advancing to a Shockley-Queisser (S-Q) value of 29.6%, with the current highest efficiency of 26.7% [2]. However, increasing power conversion efficiency (PCE) beyond the S-Q limit will lead to technological challenges and dramatically escalating costs in single-junction-based PV cells.

The perovskite solar cells (PSCs) paved the way towards cost-effective and high-performance PV technology. High absorption coefficients, high electron and hole mobilities, and long charge carrier diffusion lengths are the key parameters attributed to highly efficient PSCs [3]. With these outstanding characteristics, PSCs have demonstrated a certified 25.7% efficiency, competing with well-established c-Si solar cells [4]. Nevertheless, most of the high performance is exhibited by metal electrodes processed at high temperatures and organic hole transport materials (O-HTMs) [5]. Further, increased cost and instability issues associated with these metal electrodes and O-HTMs are still under debate [6,7].

Among different PSC configurations, HTM-free carbon electrode-based PSCs (c-PSCs) have recently been developed and exhibited excellent performance to overcome increased cost, complex fabrication, and instability issues [8]. For example, Ku et al. [9] presented an initial report in 2013 demonstrating fully printable mesoscopic c-PSC with 6.6% efficiency. Subsequently, Mei et al. [10] reported 12.8% efficient HTM-free fully printable mesoscopic PSC with carbon layer back contact and excellent stability of over 1000 h. Very recently, Zouhair et al. [11] used a 2D perovskite (passivation layer) in HTM-free c-PSC by implementing surface passivation with a 3D perovskite layer. This approach demonstrated the highest PCE of 18.5% with enhanced stability retaining more than 80% of initial efficiency following 500 h of continuous 1-sun illumination. However, with a current record of 18.5%, the efficiencies in c-PSCs are relatively lower than in PSCs equipped with metal electrodes and HTMs [12].

The S-Q limit in single-junction solar cells has been shown to be overcome by combining low and high-bandgap materials, leading to enhanced efficiency [13]. The tandem configuration is the most well-known design, wherein two or more cells are combined, each absorbing a separate part of the solar spectrum, resulting in reduced power losses and improved performance. In tandem design, the top cell with a wide-bandgap absorber utilizes high-energy photons to produce a high open-circuit voltage (Voc) with reduced thermalization losses, whereas the bottom cell with the narrow-bandgap absorbs photons of low energy to widen the photo response [14].

Numerous high-performance materials from III-V class semiconductors have been studied in tandem structures as top cells [15]. However, the high materials costs, complex fabrication techniques, and high-temperature processing have limited their application. The low-temperature solution processing and wide bandgap of perovskites mark them a potential candidate as a top cell for pairing with silicon cells in tandem arrangement [16]. Since the initial reports in 2014–15, tandem solar cells (TSCs) based on silicon/perovskite tandem configurations have gained significant interest from the scientific community and industry stakeholders [17,18]. The silicon/perovskite TSCs have recently exhibited certified efficiency of 31.3% with continuous and dedicated efforts [4].

**Fig. 1:**
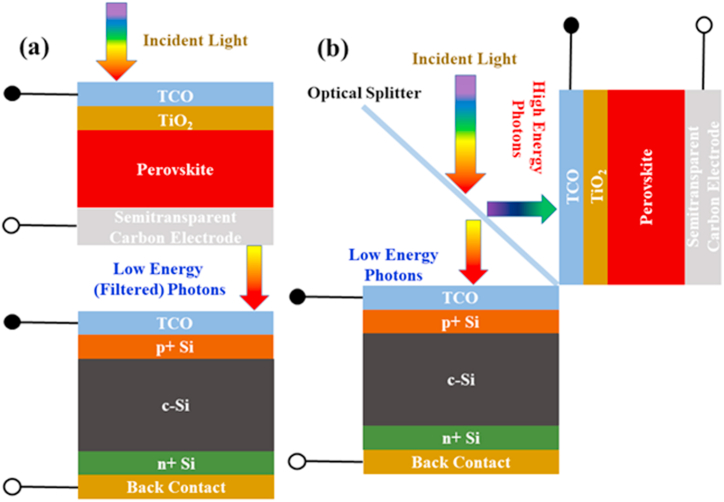
Device schematics for four-terminal (a) mechanically and (b) optically connected silicon/perovskite tandem solar cells.

In general, there are two approaches to designing TSCs, namely two-terminal (2-T) and four-terminal (4-T) tandems. Latter configurations are further classified as mechanically stacked and optically coupled [19]. The two cells in the 2-T arrangement are fabricated in series by a recombination layer or a junction [20]. The parasitic absorption is minimized in this design as only one transparent electrode is employed. On the other hand, the current matching requirement in both cells makes the fabrication process more complicated. Comparatively, the two cells in 4-T mechanically stacked TSCs are fabricated and linked independently, as shown in Fig. 1a. Similarly, in 4-T optically coupled, the two cells absorb different light spectrums separated through an optical splitter, as shown in Fig. 1b. The advantages of these designs are that they are self-contained, so if one cell fails, the other will continue to function, albeit at a reduced power level. Moreover, individual fabrication of such cells allows the user to study or analyze each cell without compromising the full device. However, the increased cost of the optical components used in the 4-T optically coupled design limits its application towards commercialization [16]. In addition, results reveal that most of the high performances from c-Si/perovskite tandem cells are exhibited by metal electrodes processed at high temperatures and O-HTMs, viz. Ag and Au, Spiro-OMeTAD, and PTAA, respectively, employed in top PSCs [21,22]. Therefore, a top cell with a low-cost and solution-processed PSC is considered desirable for cost-effective and efficient tandem cells.

In the present study, 4-T mechanically stacked and optically coupled TSCs were designed and optimized by employing SCAPS-1D (one-dimensional solar cell capacitance simulator). Low-cost, stable, and easily processed HTM-free semitransparent carbon electrode-based perovskite and c-Si PV cells were utilized as top and bottom cells, respectively. The wide bandgap (1.6 eV) multi-cation perovskite ${Cs}_{x}{\left({FA}_{0.4}{MA}_{0.6}\right)}_{1-x}{PbI}_{2.8}{Br}_{0.2}$ and a low bandgap (1.12 eV) c-Si were employed as light absorbers in the top and bottom cells, respectively. The thicknesses and doping concentrations of the top cells’ absorber are optimized for each configuration to improve performance. Although numerous studies have employed simulation programs for the modeling and optimization of silicon/perovskite TSCs [[23], [24], [25]]. The primary impetus of current research is the lack of previous research on tandem cells based on low-cost, solution-processed, stable, and highly efficient PSCs. To the best of our understanding, this is the first numerical research on TSCs based on c-PSC as a top cell. This study opens avenues towards cost-effective, stable, and highly efficient TSCs.

## 2 Methodology

**Fig. 2:**
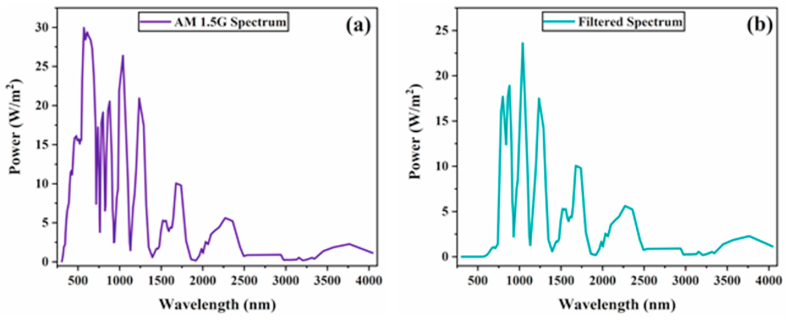
(a) Standard AM1.5G spectrum (b) filtered spectrum transmitted through top cell.

Here, the SCAPS, ver. 3.3.10 was successfully utilized to develop and optimize the four-terminal (4-T) mechanically and optically connected tandem solar cells (TSCs). The software was designed by Prof. Marc Burgelman and fellows at the Department of Electronics and Information Systems (ELIS), University of Gent, Belgium [26]. It works on the principle of three different equations: continuity, Poisson, and transport equations for electrons and holes. Before tandem cells modeling, the bottom cell was designed and calibrated with the experimental results published in Ref. [27]. In contrast, the top cell was invoked from our previously optimized study on c-PSC [28]. In both cases, the optimized results are well comparable to experimental reported findings. Table S2 presents a comprehensive assessment of our study with previously reported experimental and simulated findings based on top perovskite and bottom silicon solar cells. Here, the top cell contains a transparent conducting oxide layer based on FTO, TiO_2_ as a buffer layer, ${Cs}_{x}{\left({FA}_{0.4}{MA}_{0.6}\right)}_{1-x}{PbI}_{2.8}{Br}_{0.2}$ as a perovskite absorber and semitransparent carbon as a back electrode. The architecture of the bottom cell is composed of a moderately doped p-type c-Si layer sandwiched between highly doped p-type (p^+^) and n-type (n^+^) Si layers. The input parameters of different layers are obtained from theoretical and experimental literatures, including our previous study [23,[28], [29], [30], [31], [32], [33], [34]], and presented in Table 1. The interface defect layer (IDL) is assumed to consider interface recombination. The c-Si layer thickness in the bottom cell was considered as 230 μm thick and taken from Ref. [27]. The device parameters not specified in Table 1 are stated in Table S1. The top cell was mechanically arranged with the bottom cell to form a 4-T mechanically stacked tandem cell. While in the 4-T optically coupled design, the two cells were connected via an optical splitter, as demonstrated in Fig. 1a and b and , respectively. The 4-T mechanically stacked and optically coupled designs are termed 4-T mechanical and 4-T optical, respectively. The top cell was exposed to AM 1.5 Global spectrum in 4-T mechanical, as portrayed in Fig. 2a. The filtered spectrum (transmitted via the top cell) was used for the bottom cell. The filtered spectrum (as demonstrated in Fig. 2b) was obtained from Eq. (1) using parameters of different layers utilized in the top cell [35]. In the 4-T optical design, the two subcells were operated under their own spectrum (cut-off) separated by an optical splitter.

(1) $$S\;\left(\lambda \right)=S_{o}\left(\lambda \right)\;.\;\exp\left(\sum_{i=1}^{n}-\alpha_{{mat}_{i}}\left(\lambda \right)d_{{mat}_{i}}\right)$$

Here, S(λ), $S_{o}\left(\lambda \right)$, α, and d represent the filtered spectrum, AM1.5 Global spectrum, the absorption coefficient, and thickness of a layer, respectively.

**Table 1:** Device modeling parameters for different layers utilized in perovskite and silicon solar cells.

Parameters	Abbreviation	Unit	FTO	TiO_2_	IDL	Perovskite	Si (p+)	c-Si	Si (n+)
Thickness	–	nm	300	20	10	800 (Variable)	600	230,000	500
Bandgap	E_g_	eV	3.5	3.2	1.60	1.60	1.12	1.12	1.12
Electron Affinity	χ	eV	4	4	3.9	3.9	4.05	4.05	4.05
Relative Permittivity	ε_r_	–	9	10	6.5	6.5	11.9	11.9	11.9
CB effective density of states	N_c_	cm^−3^	2.2 × 10^18^	1 × 10^21^	1.0 × 10^17^	1.0 × 10^17^	2.819 × 10^19^	2.819 × 10^19^	2.819 × 10^19^
VB effective density of states	N_v_	cm^−3^	1.8 × 10^19^	2 × 10^20^	1.0 × 10^17^	1.0 × 10^17^	1.040 × 10^19^	1.040 × 10^19^	1.040 × 10^19^
Electron mobility	μ_n_	cm^2^/vs	20	20	2.0	2.0	1400	1400	1400
Hole mobility	μ_p_	cm^2^/vs	10	10	2.0	2.0	450	450	450
Acceptor concentration	N_A_	cm^−3^	0	–	1 × 10^13^	1 × 10^13^ (Variable)	1 × 10^20^	1 × 10^16^	–
Donor concentration	N_D_	cm^−3^	2 × 10^19^	5 × 10^19^	0	0	–	–	3 × 10^19^
Defect Density	N_t_	cm^−3^	1 × 10^16^	1 × 10^16^	1 × 10^16^	2.60 × 10^13^	–	–	–
Reference	–	–	[28,36]	[28,32]	[28]	[[28], [29], [30],^33^]	[23]	[23,27]	[23]

## 3 Results and discussion

### 3.1 Impact of the perovskite thickness

**Fig. 3:**
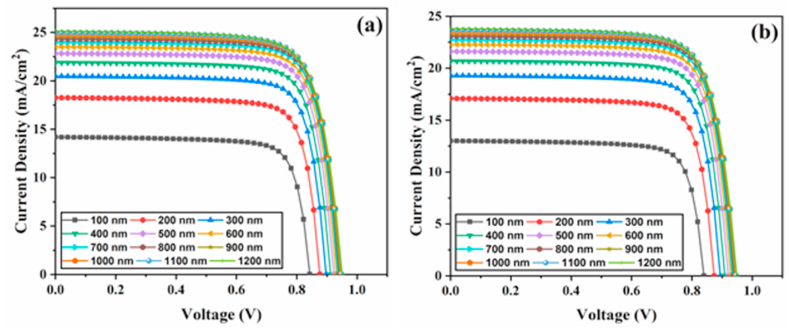
Perovskite top cell J-V parameters with various thicknesses for four-terminal (a) mechanical and (b) optical tandem solar cells.

The absorber thickness is critical to cell performance as it directly controls photon absorption. Therefore, optimizing the absorber layer thickness is essential to balance photo-induced charge carriers and recombination carriers due to their strong light absorption. Fig. 3a-b portrays sets of J-V graphs for 4-T mechanical and 4-T optical tandems with variation in top cell absorber thicknesses from 100 to 1200 nm. An increased thickness of the perovskite increases the current significantly, as illustrated in Fig. 3a-b. This improvement is associated with broad optical absorption in the light-harvesting region, which increases electron-hole pair generation.

**Fig. 4:**
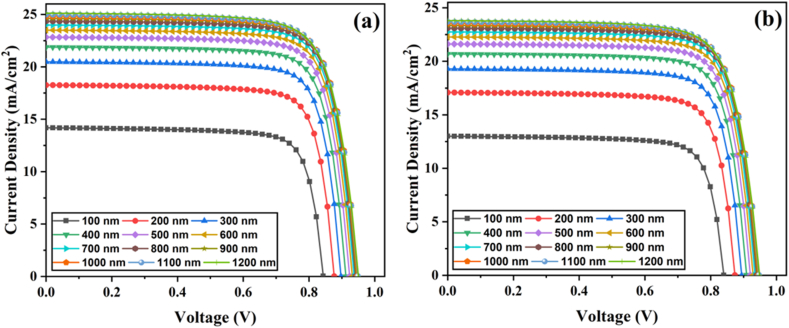
Perovskite top cell PV parameters with various thicknesses for four-terminal (a) mechanical and (b) optical tandem solar cells.

Fig. 4a-b presents the effect of different absorber thicknesses on the PV parameters for 4-T mechanical and optical cells. In both configurations, the Jsc values increase as the absorber thickness rises. At thickness values from 100 to 800 nm, J_SC_ increased significantly; however, beyond 800 nm, a marginal change can be observed. Jsc increases as the perovskite layer thickness increases, owing to more photon absorption at longer wavelengths in a thicker perovskite absorber, resulting in increased carrier generation. It can also be observed that the Jsc values obtained for 4-T optical solar cells are lower than those obtained for 4-T mechanical TSCs. This is because, in the 4-T mechanical cell, the top cell operates under the full incident spectrum, while in the 4-T optical design, the cell absorbs a portion of the incident light flux (Fig. 2b), resulting in a lower photocurrent. The Voc rises from 0.84 to 0.94 V by improving the absorber thickness to 800 nm. Then, it reaches a saturation point of 0.94 V. It is because increased thickness increases the recombination rate, which influences Voc. With increasing absorber thickness, the FF value drops dramatically. The absorber's increased thickness causes high series resistance, lowering the FF. In the top cell of the 4-T mechanical design, the PCE value rises from 9.43% to 18.06% as the thickness of the absorber rises from 100 to 1000 nm. The PCE begins to degrade beyond 1000 nm. Similarly, in the top cell of the 4-T optical design, the PCE value rises from 8.59% to 17.10% as absorber thickness increases from 100 to 1100 nm. The PCE begins to saturate beyond 1100 nm. According to our simulations, the perovskite absorber layer thickness of 1000–1100 nm is suitable for high performance. As a result, we optimized our simulated devices further by considering perovskite absorber layers with thicknesses of 1000 nm and 1100 nm for 4-T mechanical and optical architectures.

**Fig. 5:**
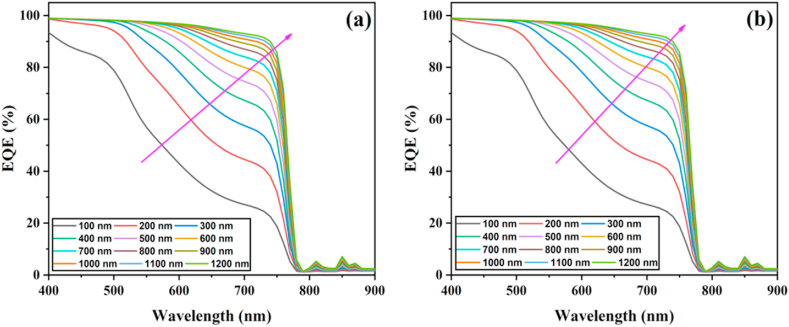
EQE graphs of perovskite top cells with various thicknesses for four-terminal (a) mechanical and (b) optical tandem solar cells.

Fig. 5a-b illustrates the variation in EQE (external quantum efficiency) for 4-T mechanical and 4-T optical TSCs. The EQE response improves considerably as the perovskite layer thickness increases, indicating a significant improvement, especially at longer wavelengths. This is because higher energy photons are absorbed close to the surface, and lower energy photons are absorbed at a nearly wide absorber layer [24]. Subsequently, increasing thickness facilitates the absorption of longer wavelength energy photons. In summary, 1000 nm and 1100 nm values are the optimum perovskite thicknesses for 4-T mechanical and 4-T optical TSCs, respectively.

**Fig. 6:**
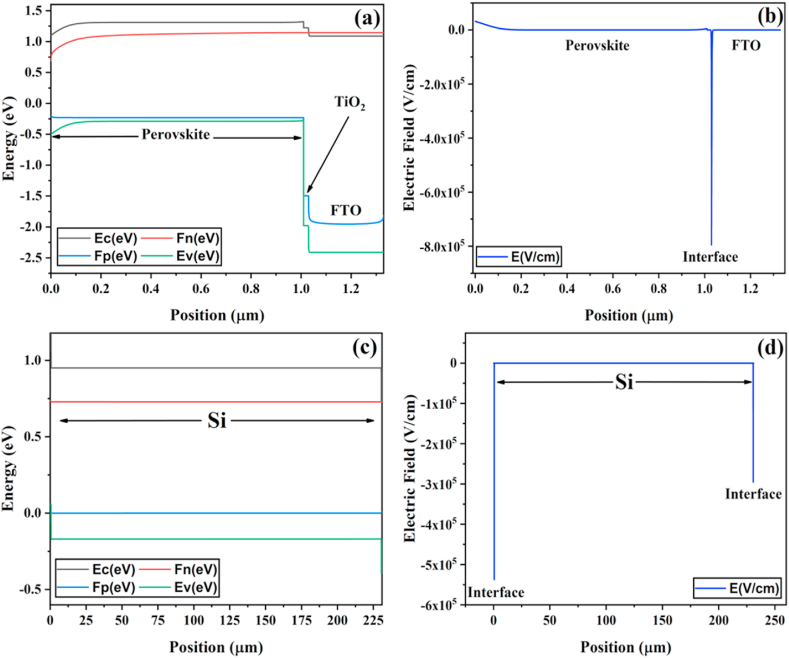
Simulated energy band diagrams and electric field distributions for (a–b) perovskite and (*c*–d) silicon solar cells.

The energy band diagrams of the proposed perovskite and silicon-based solar cells are shown in Fig. 6a and c, respectively. The low conduction band offset between the absorber and the TiO_2_ results in smooth electron transport from the absorber to the front contact (FTO), as shown in Fig. 6a. Furthermore, the higher valence band offset at the perovskite/TiO_2_ interface would create a sufficient potential barrier to prevent minority carriers (holes) from flowing toward the front contact. The electric field distribution for perovskite and silicon-based solar cells are shown in Fig. 6b and d, respectively. A strong electric field at the front surface of the PSC can be seen in Fig. 6b. This increased built-in electric potential along the absorber layer would be effective in reflecting minority carriers from the front surface and increasing the collection of photogenerated electrons and holes. This would significantly improve overall performance by reducing carrier recombination at the proposed solar cell front surface [37]. Furthermore, the strong electric field increases electrons conductivity and provides low resistance to electrons flow that are swept away from the perovskite absorber layer.

### 3.2 Impact of the perovskite doping concentration

**Fig. 7:**
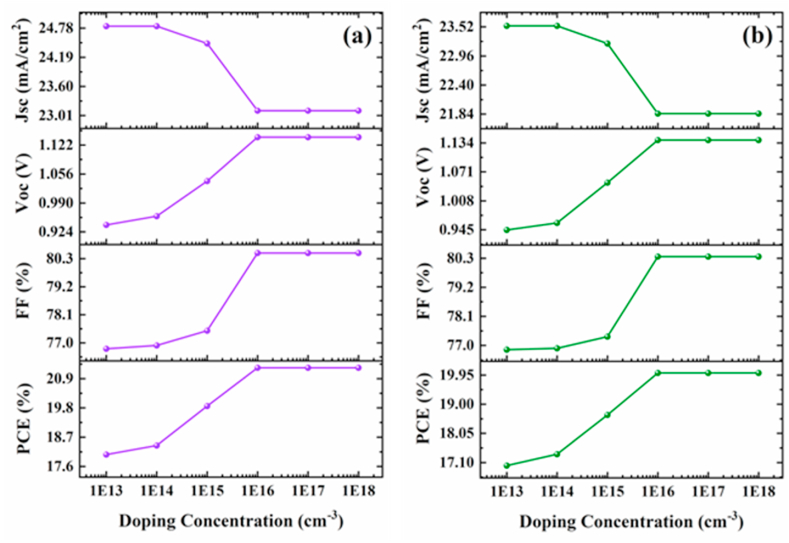
Perovskite top cell PV parameters with various doping levels for four-terminal (a) mechanical and (b) optical tandem devices.

The perovskite absorber doping concentration is important in PV cells performance. The effect of doping concentration on tandem design performance is investigated by varying the doping concentration of the perovskite top cell from 10^13^ to 10^18^ cm^−3^. Fig. 7a and b presents the PV parameters variation concerning doping concentration for 4-T mechanical and 4-T optical devices, respectively. Our results show that Jsc has a constant behavior at low doping levels; however, as doping concentration increases, Jsc declines due to device degradation induced by layer deterioration caused by the introduction of excess defect states. Results also indicate significant growing behavior in Voc and FF with changes in doping density. Initially, Voc and FF increase as doping concentrations increase, but they become independent of doping density after a certain point. Moreover, as doping concentrations rise, PCE rises rapidly, especially between 10^14^ and 10^16^ cm^−3^, from 18.39% to 21.31% and 17.37% to 20.02% for 4-T mechanical and 4-T optical arrangements, respectively. Beyond further enhancement in the doping density, the J-V parameters remain constant, and no variation is observed. Consequently, at 10^17^ cm^−3^, 4-T mechanical and 4-T optical devices exhibit maximum efficiencies of 21.3% and 20.02%, respectively. Therefore, the doping concentration of 10^17^ cm^−3^ has been selected for both 4-T mechanical and 4-T optical structures. A detail of PV parameters for both arrangements is presented in Table 2. A similar trend can be seen in both configurations, whereas the Voc and FF values are nearly identical. Also, the results show a slight decrease in Jsc and PCE values. This is because the top cell in the 4-T mechanical design absorbs the entire spectrum. In contrast, the cell in the 4-T optical design only absorbs part of the incident light, resulting in a lower photocurrent and efficiency.

**Table 2:** Perovskite top cell PV parameters with a doping concentration of 10^17^ cm^−3^ for 4-T mechanical and 4-T optical structures.

	4-T Mechanical	4-T Optical
Jsc (mA/cm^2^)	23.11	21.85
Voc (V)	1.14	1.14
FF (%)	80.52	80.37
PCE (%)	21.31	20.02

### 3.3 Design and assessment of 4-T mechanical and 4-T optical designs

In this section, the tandem structures are formed and assessed by pairing the perovskite top and the c-Si bottom cells. The perovskite cell is the same as it was optimized in the previous sections, while the calibrated c-Si cell is taken from the literature [27]. In the methodology section, the procedure for TSCs simulation is discussed. In the 4-T Mechanical design, the top cell was exposed to the AM1.5G spectrum. In contrast, the bottom cell was operated under a filtered spectrum determined using absorption coefficients and thickness of the different materials utilized in the top cell. In the 4-T optical design, the two subcells were operated under their own spectrums (cut-off), separated by an optical splitter. In 4-T mechanical and 4-T optical configurations, the top cells exhibit 21.31% and 20.02% efficiencies under the full and cut-off spectrums, respectively. At the same time, the bottom cells deliver PCE values of 7.07% and 9.32% under the filtered and cut-off spectrums, respectively. When the bottom and top cells are integrated into the tandem approach, the 4-T mechanical and optical TSCs have an overall PCE of 28.38% and 29.34%, respectively.

**Fig. 8:**
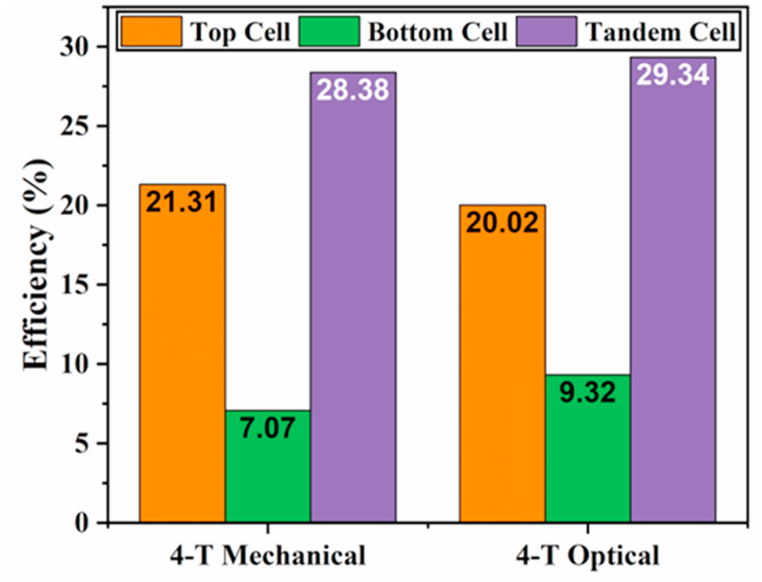
Evaluation of efficiencies for top, bottom, and tandem cells in 4-T mechanical and 4-T optical arrangements.

Fig. 8 compares efficiencies in perovskite and silicon solar cells in standalone and tandem structures for both 4-T mechanical and 4-T optical devices. According to the findings, the tandem configurations performed remarkably, with efficiencies of 28.38% and 29.34% for 4-T mechanical and 4-T optical tandem structures, respectively. Relative to the 4-T mechanical tandem cell, the 4-T optical tandem design produces a higher cumulative efficiency. The enhanced overall efficiency in 4-T optical design may be attributed to the high current density (16.13 mA/cm^2^) of the bottom cell. This is because, in the 4-T mechanical cell, the bottom cell absorbs a portion of the incident light filtered through the top cell, while in the 4-T optical design, the bottom cell operates under its own absorption spectrum, resulting in a higher photocurrent, thus enhanced overall PCE. These findings suggest that c-PSCs could be used in TSCs with c-Si to boost the performance of highly-efficient silicon PV cells. This indicates that the tandem cells modeling in this study was well performed using the optimal thickness and doping concentration of the perovskite absorber for the TSCs.

Table 3 presents the PV parameters for perovskite and silicon solar cells under different configurations and corresponding 4-T mechanical and 4-T optical TSCs. Under full incident light (standalone condition), the top perovskite cell exhibits Voc, Jsc, FF, and PCE values of 1.14 V, 23.11 mA/cm^2^, 80.52%, and 21.31%, respectively. When the same perovskite cell is exposed to the cut-off spectrum, the cell demonstrates Voc of 1.14 V, Jsc 21.85 mA/cm^2^, FF of 80.37%, and PCE of 20.02%. The Voc remains the same in both configurations, while the FF shows a marginal decrease (80.52% → 80.37%) under the cut-off spectrum. A reduction in Jsc from 23.11 to 21.85 mA/cm^2^ is due to the reduced illumination power in the cut-off spectrum. The performance of a silicon cell under full-spectrum (standalone condition) is also described in Table 3 for comparison purposes. The bottom silicon cell shows Voc of 0.69 and 0.70 V, Jsc of 12.29 and 16.13 mA/cm^2^, FF of 82.50 and 81.97%, and PCE of 7.07 and 9.32% under filtered and cut-off spectrums, respectively. The reduction in Jsc and Voc is attributed to reduced light intensity in the bottom cell. Moreover, at lower illumination, the effect of series resistance is reduced, resulting in an increase in FF values, as reported by Ref. [38]. After adding the 21.31% efficient top PSC with 7.07% silicon bottom cell, the overall efficiency of the 4-T mechanical design is up to 28.38%. Likewise, the PCE of 29.34% from the 4-T optical tandem cell is a combination of 20.02% from the top and 9.32% from the bottom cells, respectively. The overall PCE in tandem cells significantly improves efficiencies over perovskite and silicon solar cells operating independently. With PCE of 29.34%, the top cell in 4-T optical mode exhibited Voc of 1.14 V, which matches closely with the experimental value of 0.987 V [39]. Similarly, the FF of 81.97% in the bottom cell is also in good agreement with the experimental value of 80.9% [39]. This evidence supports the parameters selected for the measurements of the cells. Table S3 presents a comprehensive assessment of our study with previously reported experimental and simulated findings based on top PSCs with HTMs. The results demonstrate a considerable overall performance enhancement in tandem devices based on HTM free c-PSCs as top cells. In addition, the encouraging PCE values of 4-T mechanical and 4-T optical tandem cells in our work suggest the existence of effective, simple, stable, and inexpensive commercially viable alternatives.

**Table 3:** Comparison of PV parameters of perovskite and silicon solar cells under various configurations and corresponding 4-T mechanical and 4-T optical tandems.

Solar cells configurations	Voc (V)	Jsc (mA/cm^2^)	FF (%)	PCE (%)
Perovskite top cell under full spectrum (standalone)	1.14	23.11	80.52	21.31
Perovskite top cell under the cut-off spectrum	1.14	21.85	80.37	20.02
Silicon bottom cell under full spectrum (standalone)	0.72	38.68	78.61	22.14
Silicon bottom cell under the filtered spectrum	0.69	12.29	82.50	7.07
Silicon bottom cell under the cut-off spectrum	0.70	16.13	81.97	9.32
4-T Mechanical - combined PCE (%)				21.31 + 7.07 = 28.38
4-T Optical - combined PCE (%)				20.02 + 9.32 = 29.34

## 4 Conclusion

To summarize, the 4-T mechanically and optically connected tandem solar cells in silicon/perovskite arrangements were designed and optimized using SCAPS. In tandem configurations, a semitransparent carbon-based HTM-free multi-cation PSC top cell with ${Cs}_{x}{\left({FA}_{0.4}{MA}_{0.6}\right)}_{1-x}{PbI}_{2.8}{Br}_{0.2}$ absorber layer (Eg = 1.6 eV) was paired with c-Si (Eg = 1.12 eV) bottom cell. In a 4-T mechanically arranged design, the top cell was operated under AM 1.5 Global spectrum, while the bottom cell was operated under the filtered spectrum. In the 4-T optically connected design, each cell was operated under its own spectrum, separated by an optical splitter. The impact of perovskite thickness and doping concentrations were examined and optimized in both tandem configurations. Furthermore, the optimal values for perovskite thickness were determined as 1000 and 1100 nm for 4-T mechanically and optically arranged silicon/perovskite tandem solar cells, respectively. Similarly, the doping concentration in both configurations was found as 10^17^ cm^−3^. Under optimized conditions, 28.38% and 29.34% efficiency values were obtained in 4-T mechanically and optically connected tandem solar cells, respectively. Results reveal that the optically coupled tandem cell outperforms the 4-T mechanically stacked in terms of total efficiency. Finally, the current research points to future trends towards efficient, simple, stable, and low-cost silicon/perovskite tandem solar cells using HTM-free semitransparent carbon-electrode-based PSC.

## Author contribution statement

Ehsan Raza, Zubair Ahmad: Conceived and designed the experiments; Performed the experiments; Analyzed and interpreted the data; Contributed reagents, materials, analysis tools or data; Wrote the paper.

Fakhra Aziz, Muhammad Asif, Muhammad Qasim Mehmood, Jolly Bhadra, Noora J. Al-Thani: Analyzed and interpreted the data; Contributed reagents, materials, analysis tools or data.

## Funding statement

The article processing charges (APCs) are funded by the Qatar National Library, Qatar.

## Data availability statement

Data will be made available on request.

## Declaration of interest's statement

The authors declare no competing interests.

## Appendix A Supplementary data

2023-01-28 Supplementary Information.docx 2023-01-28 Supplementary Information.docx
