# Supplementary material for: Design and optimization of four-terminal mechanically stacked and optically coupled silicon/perovskite tandem solar cells with over 28% efficiency
Source: Heliyon. 2023 Feb 3;9(2):e13477. doi: 10.1016/j.heliyon.2023.e13477 (PMC9939591; doi:10.1016/j.heliyon.2023.e13477)
Supplement: 2023-01-28 Supplementary Information.docx [file mmc1.docx]

**Design and optimization of four-terminal mechanically stacked and optically coupled silicon/perovskite tandem solar cells with over 28% efficiency**

Ehsan Raza ^a,b^, Zubair Ahmad ^b,^*, Fakhra Aziz ^a^, Muhammad Asif ^a^, Muhammad Qasim Mehmood ^c^, Jolly Bhadra ^b^, Noora J. Al-Thani ^b^

^a^ Department of Electronics, University of Peshawar, Peshawar, 25120, Pakistan

^b^ Qatar University Young Scientists Center (QUYSC), Qatar University, 2713, Doha, Qatar

^c^ MicroNano Lab, Electrical Engineering Department, Information Technology University (ITU) of the Punjab, Ferozepur Road, Lahore 54600, Pakistan

* Correspondence: [zubairtarar@qu.edu.qa](mailto:zubairtarar@qu.edu.qa); Tel.: +974 44037729

**Supplementary Information**

Table S1: Defect parameters of different layers of simulated PSC

| **Parameters** | **FTO** | **TiO_2_** | **IDL** | **Cs_x_(FA_0.4_MA_0.6_)_1-x_PbI_2.8_Br_0.2_** |
| --- | --- | --- | --- | --- |
| **Defect Type** | Neutral | Neutral | Neutral | Neutral |
| **Electrons Capture Cross Section (cm^2^)** | 2×10^-14^ | 2×10^-14^ | 2×10^-14^ | 2×10^-14^ |
| **Holes Capture Cross Section (cm^2^)** | 2×10^-14^ | 2×10^-14^ | 2×10^-14^ | 2×10^-14^ |
| **Energetic Distribution** | Gaussian | Gaussian | Gaussian | Gaussian |
| **Defect Energy level Reference E_t_** | Above E_V_ | Above E_V_ | Above E_V_ | Above E_V_ |
| **Energy level related to Reference (eV)** | 0.6 | 0.6 | 0.6 | 0.6 |
| **N_t_ (cm^-3^)** | 1×10^16^ | 1×10^16^ | 1×10^16^ | 2.60×10^13^ |
| **Reference** | [1, 2] | [2, 3] | [2, 4] | [2, 4-7] |

Table S2: Comparison between PV parameters of simulated and experimental devices of top (perovskite) and bottom (silicon) solar cells.

| Work Mode | | Voc (V) | Jsc (mA/cm^2^) | FF (%) | PCE (%) | R_S_ (Ωcm^2^) | R_SH_ (Ωcm^2^) | Ref. |
| --- | --- | --- | --- | --- | --- | --- | --- | --- |
| Experimental | Top Cell | 0.9±0.2 | 21.45±0.65 | 69±0.1 | 13.39±0.3 | -- | -- | [5] |
| Simulation |  | 0.8953 | 21.45 | 69.06 | 13.26 | 2.35 | 1×10^30^ | [2] |
| Experimental | Bottom Cell | 0.72 | 38.68 | 78.61 | 22.14 | -- | -- | [8] |
| Simulation |  | 0.72 | 38.68 | 78.62 | 22.14 | 1.30 | 1×10^30^ | This study |

Table S3: Experimental (Exp.) and simulated (Sim.) work comparison of different PV parameters of top and bottom cells with and without HTMs and corresponding 4-T mechanically and optically connected tandem solar cells (TSCs). FTO, ETM, HTM and Au stands for front transparent oxide, electron transport material, hole transport material and gold, respectively.

| 4-T Mechanically stacked tandem solar cells | | | | | | | | | | | | |
| --- | --- | --- | --- | --- | --- | --- | --- | --- | --- | --- | --- | --- |
| Top perovskite cell configurations | | **Voc (V)** | | **Jsc (mA/cm^2^)** | | **FF (%)** | | **PCE (%)** | | **Combined PCE (%)** | **Mode** | **Ref.** |
|  |  | Top | Bottom | Top | Bottom | Top | Bottom | Top | Bottom |  |  |  |
| FTO/ETM/Perovskite/HTM/Au | With HTM | 1.06 | 0.59 | 21.52 | 15.41 | 77.48 | 76.02 | 17.7 | 7.0 | 24.7 | Exp. | [9] |
| FTO/ETM/Perovskite/HTM/Au |  | 1.04 | 0.6 | 23.3 | 16.3 | 80.6 | 82.6 | -- | -- | 27.7 | Sim. | [10] |
| FTO/ETM/Perovskite/C | Without HTM | 1.14 | 0.69 | 23.11 | 12.29 | 80.52 | 82.50 | 21.31 | 7.07 | 28.38 | Sim. | This work |
| 4-T Optical coupled tandem solar cells | | | | | | | | | | | | |
| Top perovskite cell configurations | | **Voc (V)** | | **Jsc (mA/cm^2^)** | | **FF (%)** | | **PCE (%)** | | **Combined PCE (%)** | **Mode** | **Ref.** |
|  |  | Top | Bottom | Top | Bottom | Top | Bottom | Top | Bottom |  |  |  |
| FTO/ETM/Perovskite/HTM/Au | With HTM | 0.987 | 0.728 | 10.6 | 34.9 | 71.5 | 80.9 | 7.5 | 20.5 | 28.0 | Exp. | [11] |
| FTO/ETM/Perovskite/HTM/Au |  | 0.987 | 0.602 | 18.4 | 18.5 | 81.3 | 82.7 | -- | -- | 25.6 | Sim. | [10] |
| FTO/ETM/Perovskite/C | Without HTM | 1.14 | 0.70 | 21.85 | 16.13 | 80.37 | 81.97 | 20.02 | 9.32 | 29.34 | Sim. | This work |

**References:**

1. Lin, L., et al., *Modeling and analysis of HTM-free perovskite solar cells based on ZnO electron transport layer.* Superlattices and Microstructures, 2017. **104**: p. 167-177.

2. Raza, E., et al., *Numerical modeling and performance optimization of carbon-based hole transport layer free perovskite solar cells.* Optical Materials, 2022. **125**: p. 112075.

3. Lin, L., et al., *Device Design of Doping‐Controlled Homojunction Perovskite Solar Cells Omitting HTL and Exceeding 25% Efficiency.* Advanced Theory and Simulations, 2021. **4**(2): p. 2000222.

4. Raza, E., et al., *Numerical simulation analysis towards the effect of charge transport layers electrical properties on cesium based ternary cation perovskite solar cells performance.* Solar Energy, 2021. **225**: p. 842-850.

5. Ahmad, Z., et al., *Electrical equivalent circuit (EEC) based impedance spectroscopy analysis of HTM free perovskite solar cells.* Journal of Electroanalytical Chemistry, 2020. **871**: p. 114294.

6. Mishra, A., et al., *Effect of annealing temperature on the performance of printable carbon electrodes for perovskite solar cells.* Organic Electronics, 2019. **65**: p. 375-380.

7. Minemoto, T., et al., *Numerical reproduction of a perovskite solar cell by device simulation considering band gap grading.* Optical Materials, 2019. **92**: p. 60-66.

8. Descoeudres, A., et al., *> 21% efficient silicon heterojunction solar cells on n-and p-type wafers compared.* IEEE Journal of Photovoltaics, 2012. **3**(1): p. 83-89.

9. Dewi, H.A., et al., *Four‐Terminal Perovskite on Silicon Tandem Solar Cells Optimal Measurements Schemes.* Energy Technology, 2019.

10. Kanoun, A.A., S. Goumri‐Said, and M.B. Kanoun, *Device design for high‐efficiency monolithic two‐terminal, four‐terminal mechanically stacked, and four‐terminal optically coupled perovskite‐silicon tandem solar cells.* International Journal of Energy Research, 2021. **45**(7): p. 10538-10545.

11. Uzu, H., et al., *High efficiency solar cells combining a perovskite and a silicon heterojunction solar cells via an optical splitting system.* Applied Physics Letters, 2015. **106**(1): p. 013506.
